# Strengthening the Key Features of Volumizing Fillers: Projection Capacity and Long-Term Persistence

**DOI:** 10.3390/pharmaceutics15112585

**Published:** 2023-11-04

**Authors:** Killian Flégeau, Jing Jing, Camille Vantou, Romain Brusini, François Bourdon, Jimmy Faivre

**Affiliations:** Research and Development Department, Teoxane SA, Rue de Lyon 105, 1203 Genève, Switzerland

**Keywords:** hyaluronic acid, dermal fillers, soft-tissue fillers, hydrogels, rheology, dynamic mechanical analysis, tissue projection, enzymatic degradation

## Abstract

Volumizing fillers aim to create or restore facial volume in fat layers. To provide strong tissue lifting and long-term persistence, gels are generally designed with stiff properties, characterized by a high storage modulus (G′). However, clinical evidence shows a discrepancy between high G′ and good lifting capacities, especially after skin tension has been exerted on the gel. To better explore the in vivo behavior of a gel, we first evaluated the elastic moduli of five commercial volumizers (RHA_4_, JUV_VOL_, RES_VOL_, RES_LYFT_, and BEL_VOL_) in dynamic compression mode, E′. We further developed a Projection Index score based on the rheological assessment of creep in compression to mimic skin tension-induced stress relaxation (flattening). Finally, the ability of a gel to resist enzymatic degradation was analyzed with a multidose approach. Despite similar clinical indications, volumizers exhibited distinct behaviors. RHA_4_ and BEL_VOL_ showed the highest E′ values (resistance to strain), RHA_4_, JUV_VOL_, and RES_VOL_ exhibited the greatest projection capacities, while JUV_VOL_ and RHA_4_ offered the largest persistence to enzymatic degradation. In this article, we introduce the use of the Projection Index to efficiently assess the ability of a gel to lift tissues, thus increasing preclinical models’ efficiency and reducing the need for animal studies.

## 1. Introduction

**Figure 1:**
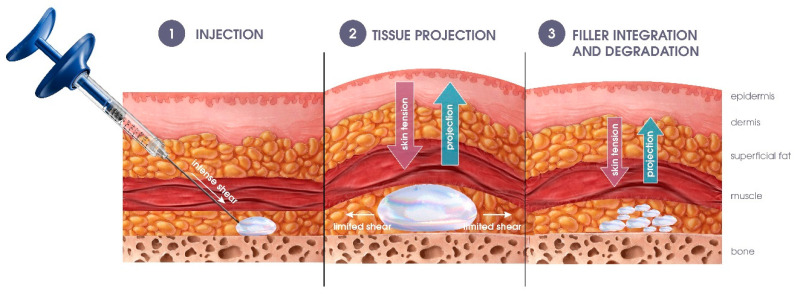
Schematics of a skin cross-section illustrate the mechanical constraints exerted by the skin on the volumizing filler. Three distinct steps were highlighted. First is the injection of quasi-elastic material through a thin-gauge needle, a step marked by a strong shear force exerted on the gel. Secondly, due to their deep injection, volumizing fillers will be mainly subjected to skin tension (mainly constant compression). Volumizers must, in turn, withstand this compression via vertical tissue projection with limited flattening. Thirdly, gel degradation occurs over time, with the opposing forces of gel persistence for a longer clinical outcome and gel degradation for safety consideration.

Hyaluronan-based (HA) fillers are viscoelastic gels specifically designed to counteract skin depressions and defects once injected at different depths of the dermis and subcutaneous tissue [1,2,3]. HA gels are mostly formed via chain etherification with the gold standard 1,4-butanediol diglycidyl ether (BDDE) in alkaline solutions [4,5,6]. Concentrations of HA and BDDE, as well as the molecular weight, can be varied to obtain hydrogels with distinct properties, adapted to correct small wrinkles or fill severe skin depressions [7]. More specifically, fillers designed for subcutaneous injections must be developed to resist horizontal (shear) and vertical (compression) forces due to skin tension and facial movements (Figure 1) [8,9,10] while maintaining skin projection [11] over extended periods of time [9]. For these reasons, volumizing fillers are generally developed as stiff, elastic materials with higher degrees of modification (MoD) and HA concentration. However, they must remain easily extrudable through thin-gauge needles (usually 27G). To evaluate their resistance to different stresses, gels are often characterized by rheology, with a specific emphasis on the elastic modulus measured in shear, G′ [12]. While the static and dynamic properties of commercial fillers have been extensively studied in shear mode, their resistance to compression and ability to project tissues remain elusive. To date, only a few studies have evaluated the behavior of gels under constant, normal compression [8,13]. As HA fillers injected into deep fat compartments mostly undergo vertical compression due to skin tension [8,13], additional information on their resistance to dynamic compression (repetitive vertical compression cycles with different intensities and probed by the elastic modulus in compression, E′) is highly desirable. In this article, we first explored the behavior of five commonly used volumizing fillers subjected to dynamic compression (via dynamic mechanical analysis, DMA) and extracted their E′ as a readout of their capacity to resist compression.

Going further, we sought to develop an analytical tool to evaluate gels’ ability to lift tissues and resist skin tension. An ideal volumizing filler should lift the tissue and maintain its thickness for as long as possible, avoiding rapid flattening that requires touch-ups and the subsequent risk of overfilling the area. We thus performed creep tests in compression that integrate the notion of time and more faithfully represent the ability of a gel to steadily resist skin-induced gel flattening. To date, only a few articles have evaluated the creep behavior of hydrogels [14,15,16], and to the best of our knowledge, our study is the first analysis performed on commercial fillers. For example, Farell et al. evaluated the creep behavior of uncross-linked HA solutions and found complete stress relaxation (flattening) within seconds, confirming the necessity of crosslinking gels to provide lifting capacities [14]. However, how the crosslinking, polymer concentration, or manufacturing process influence the gel’s ability to resist normal deformation has not yet been elucidated. To fill this gap, a new Projection Index score based on the compression creep profile of the gels was introduced to perform comparison analyses between fillers. As the lifting capacities of a gel will decrease upon its progressive resorption, we finally evaluated gels resistance to enzymatic degradation as an estimation of their durability in vivo following injection.

In this article, we aim to broaden the analytical tools that can be employed to characterize soft tissue volumizers. While oscillatory rheology remains the gold standard, our study introduces for the first time the use of the Projection Index to evaluate the ability of a gel to lift tissues over time. While previous tests only reported instantaneous measures of gels’ mechanical properties, our study is the first evaluation of hydrogels’ resistance to skin tension as a function of time. Our motivation for this study was to provide in vitro tools to explore gel behavior and help researchers mitigate any performance or safety concerns with their product before clinical evaluations. Ultimately, such parameters should reduce the need for animal studies and improve patient outcomes.

## 2. Materials and Methods

### 2.1. List of Investigated Fillers

The list of investigated fillers is compiled in Table 1. All these gels are intended to be injected deeply into fat compartments for the correction of severe wrinkles or the creation of volume.

### 2.2. Determination of the Dynamic Elastic Modulus in Compression Mode

The viscoelastic parameters of the gels were determined by oscillatory compression deformations from 0.1 to 10% (1 Hz, 25 °C) with a parallel plate geometry (40 mm anodized aluminum, TA Instruments, New Castle, DE, USA) on a DHR2 rheometer (TA Instruments, New Castle, DE, USA). For each measure, 1.5 mL of filler was deposited on the center of the Peltier plate. The gap was set to 1 mm, and the experiment started. This test measures the evolution of the elastic and viscous moduli in compression, respectively, E′ and E″, over the range of deformations. The Linear Viscoelastic Region (LVER) of each product, representing the range of deformation for which E′ is maintained constant (maintenance of at least 90% of the initial E′ value measured in quasi-static conditions), was also determined.

### 2.3. Projection Index Measurement

The volumizing capacity test was performed at a constant compression force of 2 N (1600 Pa) for 1 h at 25 °C using a parallel plate geometry (40 mm anodized aluminum, TA Instruments, New Castle, DE, USA) with a DHR2 rheometer. Based on preliminary experiments, the value of 2 N was selected as the minimal force required to induce progressive flattening of the tested gels. One gram of gel was deposited on the Peltier plate, and a conditioning step consisting of a vertical compression of the sample until reaching a 700 µm gap was performed. The recording of gel thickness over time was started when the normal force reached 2 N. A gap change-down limit was set at 200 µm to avoid direct contact between the plates during the experiment. To determine the gel thickness at equilibrium, d_∞_, a generalized Maxwell model for viscoelastic materials was used. The Projection Index (P_Idx_ in %) was then determined using Equation (1):

(1) $$P_{Idx}(\%)=\frac{d_{\infty }}{d_{initial}}\times 100$$

Throughout the experiment, gels were covered with a solvent trap to avoid water evaporation and gel drying. In parallel with this analysis, disks of porcine adipose tissue extracted from the skin of pig flanks were similarly tested. Tissues were provided and prepared by a local slaughterhouse 4 h after animal sacrifice (Loëx, Bonne, Switzerland).

### 2.4. Persistence of Volumizers Assessed by Enzymatic Degradation

The protocol used for the multidose hyaluronidase degradation of volumizers was adapted from Flégeau et al. [18]. This protocol was previously developed to assess the degradation of soft tissue fillers in contact with hyaluronidase by rheological time sweeps and real-time measurement of G′. Briefly, 50 μL of the Hylenex solution (150 USP U·mL^−1^, Halozyme Therapeutics, San Diego, CA, USA) were poured in an empty syringe and mixed with 1 mL of gel using a Luer-Lock connector. The gel was then deposited onto the DHR2 rheometer equipped with a cone (40 mm, 1°) plate geometry and allowed to equilibrate for 1 min at 37 °C before starting rheological time sweeps (0.1% strain, 1 Hz). Every 5 min, the upper geometry was raised, and 50 µL of fresh Hylenex solution was added to the gel before starting another time sweep procedure. This set-up was repeated until reaching near-complete degradation of the volumizers, set at G′ < 30 Pa.

### 2.5. Data Presentation

Experiments were conducted in triplicate unless otherwise specified. Results are presented as mean ± SD.

## 3. Results

### 3.1. Viscoelasticity Characterization of Volumizers in Compression Mode

**Figure 2:**
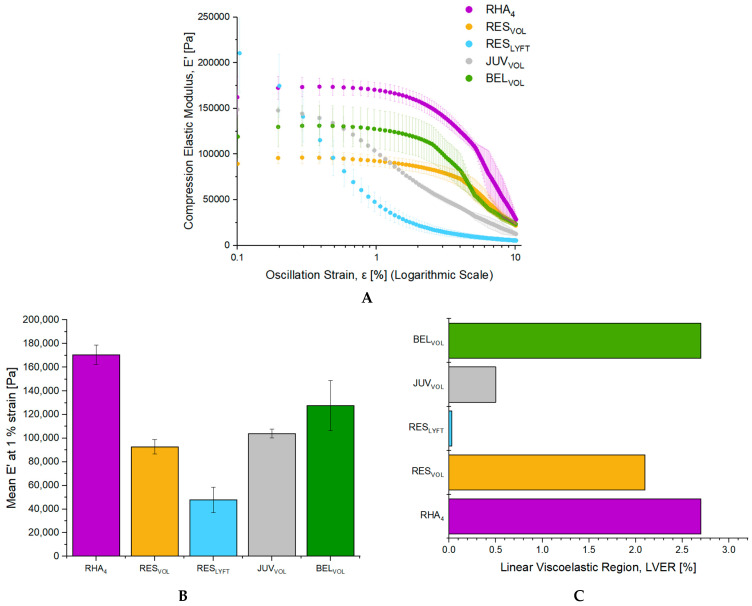
Viscoelastic characterization of the volumizers in compression mode. (**A**) Evolution of the compression elastic modulus, E′, as a function of the applied oscillatory compression deformation for the investigated fillers. The deformation range was comprised between 0.1–10% at a frequency of 1 Hz at 25 °C. (**B**) Elastic modulus E′ at 1% strain of the volumizers. (**C**) Linear Viscoelastic Regions (LVER) of the investigated fillers. LVER was determined as the oscillatory strain value for which the elastic modulus E′ retains above 90% of its initial value at 0.1% strain. All results are presented as mean ± SD, *n* = 3.

To experimentally assess the effect of dynamic compression on the fillers’ mechanical properties, the evolution of the elastic modulus E′ in compression over a deformation range of 0.1–10.0% was evaluated by rheology (Figure 2A). The shear compressive moduli at 1% strain and the linear viscoelastic region (LVER) were extracted from the curves (Figure 2B,C). Even at modest deformations (<1% strain), some fillers, including RES_LYFT_ and JUV_VOL_, showed a drastic drop in their E′ modulus. RHA_4_ showed the highest E′ value at 1% with 170,482 ± 8154 Pa, followed by BEL_VOL_, JUV_VOL_, RES_VOL_, and RES_LYFT_, with E′ values of 127,584 ± 21,203 Pa, 103,979 ± 3788 Pa, 92,746 ± 6185 Pa, and 47,819 ± 6185 Pa, respectively (Figure 2B). Concomitantly, the capacity of fillers to resist compressive strain greatly varied between gels. While BEL_VOL_, RES_VOL_, and RHA_4_ could resist deformation exceeding 2.0% of their initial height, JUV_VOL_ could only resist 0.5% strain, while RES_LYFT_ showed no LVER with a constant drop in its mechanical properties (Figure 2C).

### 3.2. Volumizing Capacities and Projection Index Measurement

**Figure 3:**
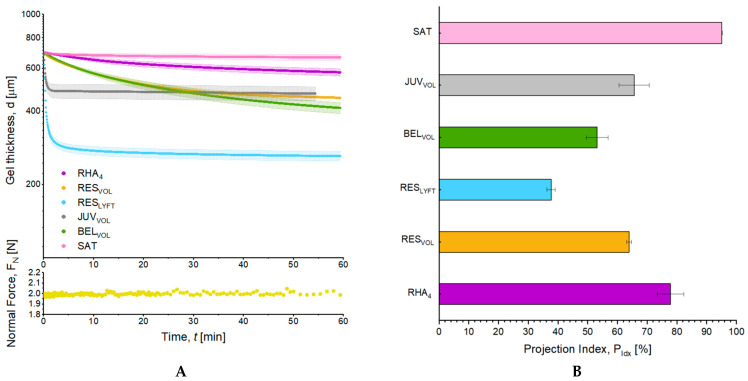
(**A**) Creep tests performed on the volumizing fillers and pig subcutaneous adipose tissue (SAT) monitored the decrease in gel thickness over 1 h at a constant normal force of 2 N. (**B**) Projection Index at equilibrium, P_Idx_, values of the fillers, and SAT calculated from a generalized Maxwell model. All gel results are presented as mean ± SD, *n* = 3.

The Projection Index (P_Idx_) of the investigated fillers was developed to measure their capacity to lift tissues while enduring skin tension. For this purpose, creep compression tests were performed to monitor the evolution of gel thickness over time under a constant normal force of 2 N, corresponding to a pressure of 1600 Pa or the application of a mass of 16 g over 1 cm^2^ of skin (Figure 3A). The configuration (normal force, gap, and geometry) was optimized to avoid immediate flattening of the least resistant fillers. For comparison, similar tests were performed with porcine subcutaneous skin tissue to evaluate the natural propensity of fatty tissues to resist compression. All fillers showed a progressive decrease in thickness over time, yet with distinct profiles (Figure 3A). Two distinct behaviors were distinguished, with RES_LYFT_ and JUV_VOL_ gels’ thickness instantly dropping under stress and reaching a near-plateau in less than 2 min. More specifically, RES_LYFT_ lost half of its initial thickness in less than 1 min. Conversely, RHA_4_, RES_VOL_, and BEL_VOL_, but also the porcine adipose tissue, showed a progressive decrease in their initial thickness over a 1-h period, with RHA_4_ demonstrating the closest behavior to porcine skin. To determine the gel thickness at equilibrium, a generalized Maxwell model for viscoelastic materials was implemented, and the Projection Index was calculated according to Equation (1) (Figure 3B). This parameter probes the filler’s final thickness at equilibrium and outlines its capacity to project tissues and maintain its height over time. RHA_4_, RES_VOL_, RES_LYFT_, BEL_VOL_, and JUV_VOL_ showed a P_Idx_ of 77.8 ± 4.5, 63.9 ± 0.8, 37.7 ± 1.4, 53.2 ± 3.7, and 65.6 ± 5.1%, respectively. Comparatively, porcine adipose tissue showed a P_Idx_ of 90%. Thus, RHA_4_, JUV_VOL_, and RES_VOL_ showed the best ability to resist deformation, with RHA_4_ showing the highest and most similar projection capacity to porcine adipose tissue.

### 3.3. Resistance of Volumizers to Enzymatic Degradation

**Figure 4:**
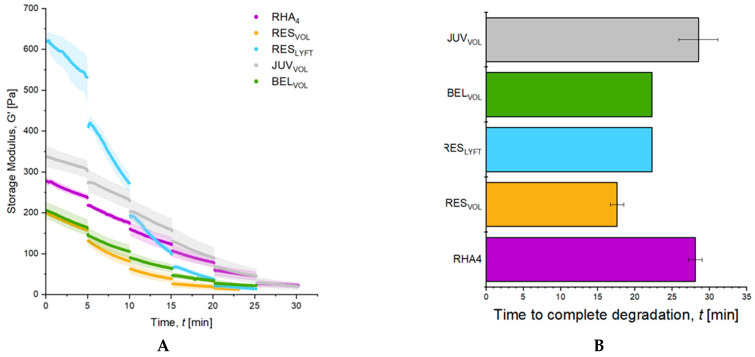
Multidose enzymatic degradation of volumizers. (**A**) Evolution of the storage modulus G′ of different volumizers over time in contact with repetitive doses of hyaluronidase; (**B**) time required to reach near-complete degradation, set at 30 Pa. All results are presented as mean ± SD, *n* = 3.

To evaluate and compare the resistance of volumizers to enzymatic degradation, a multidose degradation assay with Hylenex human recombinant hyaluronidase was performed and monitored by rheological time sweeps at 37 °C (Figure 4). Every 5 min, a new injection of 50 µL of hyaluronidase was performed until reaching near-complete degradation. All gels showed a progressive loss of their elastic moduli over time, indicative of gel degradation (Figure 4A). All gels but RES_LYFT_ showed similar degradation profiles, with a relatively linear degradation pattern. Although having the highest initial elastic modulus, RES_LYFT_ gels showed a drastic reduction in their mechanical properties following the first two hyaluronidase injections. The time required to reach a G′ of 30 Pa (used as a reference for near-complete degradation) was next calculated and found to be 28.5 ± 2.6, 22.3 ± 0.0, 22.3 ± 0.0, 17.6 ± 0.9, and 28.1 ± 0.9 min for JUV_VOL_, BEL_VOL_, RES_LYFT_, RES_VOL_, and RHA_4_, respectively (Figure 4B). Among the tested fillers, JUV_VOL_ and RHA_4_ showed the longest persistence. Hence, all gels showed the ability to be degraded, yet with distinct resistance to enzymatic cleavage.

## 4. Discussion

Developing volumizing fillers with strong mechanical properties to project tissues and slow down—but not prevent [19]—degradation while being injected through a thin needle is challenging. Only a narrow range of HA and crosslinker concentrations are effective in formulating a gel, and one could easily think that all volumizing fillers would unveil similar behaviors. Thus far, very distinct rheological and physicochemical properties are obtained when testing commercial fillers. These differences, mainly originating from manufacturing technologies, deeply impact clinical outcomes, thus motivating this comparative study. Today, the shear elastic modulus (G′) is a common way to assess gel stiffness, describing the ability of a gel subjected to lateral forces to store energy without irreversibly deforming and being reported for quasi-static stresses. However, gels for supraperiosteal injections or injections into deep fat are mainly subjected to vertical compression stresses, mostly muscular contractions and upper skin layer tension [8,11,13]. To better reproduce native conditions, we evaluated the hydrogel’s elastic modulus, E′, describing the gel’s stiffness in dynamic compression. A broad range of strains was applied to cover the full spectrum of facial dynamic expressions, not only describing gel behavior in nearly static conditions as it is commonly reported for G′ values—yet not representative of real clinical conditions. Despite similar indications, clear differences were obtained between gels, with RES_LYFT_ being unable to withstand even minor deformations (0.1% strain) while the other fillers remained stable in more dynamic environments, potentially providing different clinical features once injected. As the goal of soft tissue volumizers is to project tissues, we further aimed to assess fillers’ lifting abilities. While the G′ and E′ moduli provide some understanding of the gel mechanical resistance at rest, they do not probe the time-dependent effect of skin tension on the hydrogel. To date, only animal models, in vivo 3D imaging, and visual scales in patients provide the final assessment of a gel lifting capacity during the preclinical and clinical phases [20,21]. Although there is tremendous interest in facilitating upstream innovation and pre-clinical developments, in vitro studies evaluating tissue projection are yet to be developed. To capture the time-dependent effect of skin tension on the gel, we performed a creep test in compression, evaluating how gels would flatten under a constant compression force. The vertical pressure applied roughly corresponded to the pressure of a pencil placed on 1 cm^2^ of skin. As a reference standard, we evaluated the creep profile of porcine adipose tissue where volumizing fillers are generally injected, i.e., in fat compartments. Although it is stiffer than human skin [22], porcine skin is considered the most accurate model for human skin and provides valuable insights on the behavior of soft tissues under compression. Interestingly, the ability of a gel to resist compression was not correlated to the reported G′ values, nor was it to the HA concentration or MoD [12,13,23]. RES_LYFT_ and JUV_VOL_ almost instantaneously deformed until reaching a descending plateau. The extent of stress relaxation was maximal for RES_LYFT_ and more limited for JUV_VOL_ and was dependent on the initial force applied. Conversely, BEL_VOL_, RES_VOL_, and RHA_4_ showed a more progressive stress relaxation profile, as commonly observed for viscoelastic tissues [16,24,25]. We hypothesized that these different profiles could originate from their variations in formulation and texture. Gels with poorly interconnected crosslinked particles—e.g., NASHA Technology—tend to promptly relax through rapid energy dissipation originating from particle rearrangement [26]. Contrarily, gels with a more entangled and homogeneous network mostly relax through chain rearrangement, thus slowing down stress relaxation [27]. As observed with the porcine skin, viscoelastic tissues tend to progressively relax through collagen fiber rearrangement, water movements, and free HA non-covalent interactions [24,28]. Still, this relaxation profile was found to be slower than all the tested gels, showing the ability of fat tissue to resist deformation. Interestingly, RHA_4_, RES_VOL_, and BEL_VOL_ showed the closest behavior to porcine skin with the highest Projection Indices. Although manufactured with different processes, the aforementioned gels share a more “monophasic” aspect than the “particulate” gels. Several parameters, like the size of the particles and the cohesivity of the gels, partly explain these differences [29]. In addition, high-molecular-weight polymers are known to stress–relax more slowly than their low-molecular-weight counterparts [16]. Hence, manufacturing processes that better preserve the molecular weight of HA—as already demonstrated for RHA_4_ [12]—might have a competitive advantage over other technologies that more severely impact the polymer.

HA fillers also need to support tissue lifting as long as possible to maintain a suitable clinical duration for patient satisfaction, especially in the case of volumizing fillers. Conversely, fillers must remain naturally resorbable and degradable through the action of exogenous hyaluronidase in emergency cases [30]. To evaluate their ability to resist degradation, a multidose degradation assay was finally performed based on a previously published protocol [18]. In vitro, all gels could be completely degraded using multiple injections of small volumes of hyaluronidase, with JUV_VOL_ and RHA_4_ showing the highest persistence and RES_VOL_ the fastest degradation. This is in line with a recent publication demonstrating that longer clinical persistence of subcutaneously injected physical hydrogel in mice has been shown to correlate with reduced creep, or, in other words, with a higher Projection Index [31]. Again, no direct correlation with the MoD or HA concentration could be established, highlighting the central role of the manufacturing process on the final gel properties. It is noteworthy that other factors, such as phagocytosis and reactive oxygen species, contribute to gel resorption [32]. For example, La Gatta et al. assessed the degradation of volumizers in contact with reactive oxygen species (ROS) [23]. While RHA_4_ and RES_LYFT_ showed progressive degradation rates, JUV_VOL_ quickly degraded upon exposition to the ROS. Thus, hydrogels may have different sensitivity to different degradation mechanisms.

Potential limitations to this in vitro study are related to the direct translation of experimental results into clinical evidence. It is important to note that even for a class of similar products—herein volumizing fillers—with similar indications, fillers are not uniformly employed in the same exact layer, with the same injected volume, and with the same technique of injection, thus making direct clinical comparisons difficult. As an illustration, RHA_4_ has a unique ability to be used both as a dynamic volumizing filler in the superficial fat compartments and for supraperiostal injections [11]. Comparatively, most static volumizing fillers can only be injected close to the bone [11]. This in vitro study is thus designed to provide guidance for efficient upstream innovation.

## 5. Conclusions

In this article, we report for the first time a Projection Index directly evaluating the ability of a filler to vertically lift tissue over time. While no clear relationship between hydrogel composition and projection capacities could be highlighted, HA molecular weight clearly appeared as a hidden parameter in the equation. As gels are crosslinked in relatively harsh conditions using BDDE, our study clearly emphasizes the benefits of limiting HA degradation during the manufacturing process to preserve HA intrinsic biomechanical properties and more faithfully adapt to real-life conditions, i.e., provide high mechanical resistance to stresses (E′), high projection capacity (P_Idx_), and high product persistence. Future investigations should evaluate how human subcutaneous tissue deforms under compression and whether HA fillers can partially restore the native mechanical behavior of elder skins.

## 6. Patents

The patent application FR2208223 has been filed for the Projection Index presented in this article.

## Figures and Tables

**Table 1:** List of the volumizing fillers investigated in this study.

Filler	Manufacturer	Technology	HA Concentration (mg/mL) ^a^	MOD (%) ^b^	Shear Elastic Modulus G′ (Pa) ^c^	Injection Forces (N) ^d^	Batch References
Belotero^®^ Volume (BEL_VOL_)	Merz, Plan-les-Ouates, Switzerland	Cohesive Polydensified Matrix	26	8	198	19.5 (TSK 30G × ½″)	557104/1
Restylane^®^ Volume/Contour (RES_VOL_)	Galderma, Uppsala, Sweden	Optimal Balance Technology/XpresHAn	20	7	172	10.3 (TSK 27G × ½″)	17936-1
Restylane^®^ Lyft (RES_LYFT_)	Galderma, Uppsala, Sweden	NASHA	20	1	855	21.7 (29G × ½″TW)	15085-1
Juvéderm^®^ Voluma (JUV_VOL_)	Abbvie (Allergan), Pringy, France	Vycross	20	6	312	8.1 (TSK 27G × ½″)	VB20A60547
Teosyal^®^ RHA 4 (RHA_4_)	Teoxane, Geneva, Switzerland	Preserved Network	23	4	260	9.0 (TSK 27G × ½″)	TPUL-194921-A

^a^ as indicated in the dermal filler package insert. ^b^ as determined by internal ^1^H-NMR analyses. ^c^ as determined internally by rheological stress sweeps according to Faivre et al.’s procedure [12]. ^d^ based on the protocol of Brusini et al. [17] and using the syringes and needles provided by the manufacturer.

## Data Availability

The data presented in this study are not publicly available due to intellectual property.
